# Ecophysiological Variability of *Alnus viridis* (Chaix) DC. Green Alder Leaves in the Bieszczady Mountains (Poland) †

**DOI:** 10.3390/plants10010096

**Published:** 2021-01-06

**Authors:** Andrzej Skoczowski, Magdalena Odrzywolska-Hasiec, Jakub Oliwa, Iwona Ciereszko, Andrzej Kornaś

**Affiliations:** 1 Institute of Biology, Pedagogical University of Cracow, Podchorążych 2, 30-084 Krakow, Poland; andrzej.skoczowski@up.krakow.pl (A.S.); jakub.oliwa@awf.krakow.pl (J.O.); 2 Faculty of Biology and Agriculture, University of Rzeszow, Ćwiklińskiej 1, 35-601 Rzeszów, Poland; maglukhas@wp.pl; 3 Department of Chemistry and Biochemistry, Institute of Basic Sciences, University of Physical Education, Jana Pawła II 78, 31-578 Kraków, Poland; 4 Faculty of Biology, Department of Plant Biology and Ecology, University of Bialystok, Ciołkowskiego 1J, 15-245 Bialystok, Poland; icier@uwb.edu.pl

**Keywords:** ^13^C discrimination, chlorophyll *a* fluorescence, green alder, ^15^N discrimination, JIP-test, reflectance

## Abstract

*Alnus viridis* (Chaix) DC., green alder, is a fast-growing shrub that grows expansively in the European mountainside. In Poland, *A. viridis* naturally occurs only in the Bieszczady Mountains (south-eastern part of the country), above the upper forest border. In this study, we assessed the potential of green alder to expand in post-farming areas in the Bieszczady Mountains. We investigated the effects of topographical, climatic, and edaphic characteristics of four various study sites on the physiological and morphological properties of *A. viridis* leaves in order to answer the question whether the growth of plants in lower positions improves their physiological condition to such an extent that it increases the species invasiveness. This is the first comprehensive ecophysiological study of this species to be carried out in this part of Europe. The photochemical efficiency of PSII, the chlorophyll content, and leaf ^13^C and ^15^N discrimination were analyzed. On the basis of leaf radiation reflection, coefficients such as reflectance indices of anthocyanins, carotenoids, flavonoids (ARI2, CRI1, FRI), photochemical index of reflection (PRI), and the water band index (WBI) were calculated. We observed favorable physiological effects in *A. viridis* plants growing in locations below the upper forest border compared to plants growing in higher locations. As a result, *A. viridis* may become an invasive species and disturb the phytocoenotic balance of plant communities of the altitudinal zones in the Polish Western Carpathians.

## 1. Introduction

*Alnus viridis* (Chaix) DC., known as a green alder, is a shrub belonging to the birch family (*Betulaceae*). *A. viridis* is a heliophilous species; it is also resistant to harsh mountain conditions, including low temperatures. White et al. [1] argued that *A. viridis* plays an important role as a pioneer species because it successfully colonizes areas after strong environmental disturbances. *A. viridis* is a woody boreal species with an Alpine-Central European area of diffusion, common at the tree line and at high latitudes. It is found in the mountains of Central Europe, specifically in France, Switzerland, Italy, and Germany to Ukraine, Romania, and Bulgaria. In Poland, green alder reaches the north-western border of the Carpathian range and has the character of the Eastern Carpathian and subalpine species [2]; its natural sites occur only in the Bieszczady Mountains (south-eastern part of Poland) on the mountain slopes. *A. viridis* growing in the Bieszczady influences plant communities that prefer moderately moist habitats [3]. Despite the fact that green alder prefers moist soil on sheltered northern slopes, it copes well in quite dry and difficult environments, for example, on rock rubble or sunlit mountain slopes [3]. *A. viridis* is a rare example of a woody plant that is able to bind free N_2_, thanks to symbiosis with *Frankia alni* (Gram-positive, actinomycete filamentous bacterium) present in its root nodules [4,5,6]. The occurrence of *A. viridis* in natural localities in Poland is associated with fresh and humid habitats above the upper forest border. Lower positions, on the other hand, are thought to be a result of expansion into former farmland and grazing areas [2]. Numerous studies indicate that *A. viridis* is a very expansive species in subalpine grasslands, currently facing major environmental changes, especially in the French and Swiss Alps [7,8,9]. The rapid spread of green alder there has a negative impact on the biodiversity of plant communities [7,8].

Mountain plants are exposed to many simultaneous stress factors. These factors are mainly abiotic, including low temperature, intensive irradiation with an increased proportion of ultraviolet (especially UV-B, 280–320 nm), and a deficit of water and mineral nutrients. Temperature and radiation often change very fast, both daily and during the growing season. In addition, mechanical factors such as strong wind, snow/ice, or rock movements can injure plants [10]. In the top parts of the Bieszczady Mountains (1100–1400 a.s.l.), the daily temperature of the air is usually a few degrees lower than in the valleys (500–900 a.s.l.). The average annual temperature varies from about 4 °C in higher parts of the mountains to higher than 7 °C in the north-western part [11]. The intensity of UV-B radiation in the mountains increases with rising elevation (about 14–18% per 1000 m) [12]. Plants growing above the upper forest border are thought to be more tolerant to UV-B. For many other plants, exposure to strong solar radiation, in combination with low temperatures, is particularly disadvantageous and could be harmful [10]. Under the influence of such stressors, there is a disturbance in the equilibrium between the light and dark phases of photosynthesis. As a consequence, this can lead to damage of photoreceptors and photosystems (mainly photosystem II (PSII)) in the chloroplasts of stressed leaves [13,14]. The possibility of ground frost occurrence in the highest part of the Bieszczady Mountains was confirmed even in July, during the middle of the growing season [11]. Such an event can be very injurious to plants because it can lead to leaf tissue damage, especially of young leaves, and to morphological as well as physiological damage of photosynthetic apparatus [10,15]. An extremely important factor is water availability in soil, which affects soil moisture and determines life processes such as growth, reproduction, and survival of mountain plants. Annual rainfall in the Bieszczady ranges from 700 to 1100 mm. Paradoxically, water available in the form of fog could also damage leaves, especially of plants growing above 1000 m a.s.l. [16]. An unfavorable factor for mountain plants that causes mechanical stress is wind. In the Bieszczady Mountains, the southerly winds from the nearby Pannonian areas (the Hungarian Plain) have a very drying affect, which influences the landscape and vegetation of the mountainous meadows. It was found that plants growing on the northern slopes are less exposed to this wind as a stressor. However, on the northern slopes, plants are exposed to another unfavorable mechanical factor in the form of snow cover. Snow plays an unhelpful role by blowing, above the upper forest border, and melting slowly on the northern slopes that are sheltered from the wind. On the one hand, snow cover protects the soil from deep freezing and thus protects wintering plants against low temperatures. On the other hand, snow that refuses to melt negatively affects plant life, e.g., by shortening the growing season. In the top parts of the Bieszczady Mountains, snow appears on average as early as in mid-October and melts in mid-May [11].

Changes in the mountain ecosystem similar to those taking place in the Alps (e.g., cessation of sheep grazing) may cause extensive colonization of the lower altitudinal zonation by *A. viridis* also in the Polish part of the Carpathians where the elevations are not so significant. For this reason, the aim of our study is to determine whether the growth of plants in positions below the upper forest line improves their physiological condition (in relation to plants from higher localities) to such an extent that it increases the potential invasiveness of the species, which may result in disturbance of the phytocenotic balance of plant communities in post-agricultural areas. For this purpose, it was determined whether the elevation and nature of the habitat (including exposure to stressors) influenced leaf morphology, photosynthetic apparatus, stable isotope composition (^13^C and ^15^N) and the content of photosynthetic and photoprotective pigments in *A. viridis* leaves.

## 2. Results

### 2.1. Leaf Morphology

The results of *A. viridis,* the appearance of the bush and leaf morphology, varied depending on the study site and locality (Supplementary Table S1, Figures S1 and S2). The highest basic morphological parameter values, i.e., surface area, length, width, and perimeter, were characterized by *A. viridis* leaves from the lowest localities, i.e., 568 m and 980 m a.s.l. (Łobozew and Przełęcz Wyżna, respectively) (Table 1). However, the values of these parameters for *A. viridis* leaves growing at 980 m a.s.l. were significantly lower than those growing at 568 m a.s.l. The leaves of shrubs growing in a higher locality—1215 m a.s.l. (Połonina Wetlińska) and 1320 m a.s.l. (Tarnica)—showed the lowest values for all parameters, statistically different from the lower elevations (Table 1).

The specific leaf weight (SLW) coefficient was significantly different depending on the locality elevation (Table 1). The lowest SLW value was observed for *A. viridis* leaves at an elevation of 568 m, while the highest was at 1215 m a.s.l. Intermediate SLW values were found at 980 m, while at 1320 m the value of SLW was slightly lower than that at 1215 m a.s.l.

### 2.2. Chlorophyll (Chl) Content in Leaves

The total content of Chl (Chl *a* + *b*) in *A. viridis* leaves at the beginning of summer in the Bieszczady Mountains (July) was lowest in the lowest locality (568 m a.s.l.). At the highest localities (1320 and 1215 m), the content of Chl was similar, although significantly lower compared to that at 980 m a.s.l. (Table 2). In autumn (September) the total Chl content was significantly lower compared to July at the lowest elevations (568 m and 980 m a.s.l.) and it did not significantly change when compared to 1215 m and 1320 m a.s.l. In leaves from all localities, the total Chl as well as the Chl *a* content was higher in the leaves of *A. viridis* at the beginning of summer than in autumn (Table 2). However, the content of Chl *b* in *A. viridis* leaves was lower in July compared to September, except for the site at 980 m a.s.l. In September, the leaves of shrubs growing in lower localities had significantly less Chl *b* than those from the higher ones. Chl *a*/*b* ratios were significantly higher in the summer than in the autumn, except for the locality at 980 m a.s.l. In addition, the higher localities were characterized by a lower value of the ratio Chl *a*/*b* in relation to those located lower, which was particularly obvious during autumn months (Table 2).

### 2.3. The Leaf Isotopic Signature: ^13^C and ^15^N

The analysis of ^13^C discrimination showed significant differences between the studied shrubs (Table 3). The largest negative δ^13^C discrimination values (−30.78‰) indicative of optimal growth conditions, were found in *A. viridis* growing near the village of Łobozew at the lowest locality (568 m a.s.l.). At other field sites, Przełęcz Wyżna (980 m a.s.l.), and Tarnica (1320 m a.s.l.), the differences in δ^13^C values were smaller (Table 3). The pattern of changes in the δ^15^N discrimination value is very similar to the δ^13^C profile (Table 3). The largest negative values were recorded at the locality in Łobozew 568 m a.s.l. (Table 3).

### 2.4. Analyses of Chl a Fluorescence

**Figure 1:**
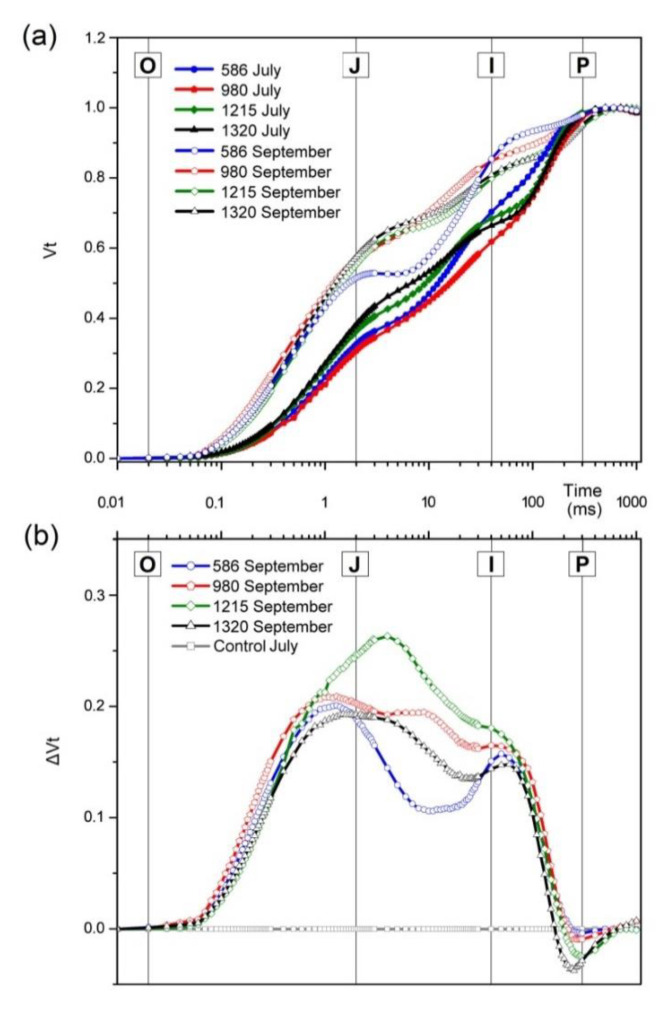
Normalized fluorescence induction curves (Vt) of Chl *a* fluorescence of green alder (*Alnus viridis*) leaves recorded in July (filled points) and September (empty points) in localities at different elevations, 586, 980, 1215, and 1320 m a.s.l. (**a**) The differential fluorescence curves (ΔVt) obtained by subtracting the Chl *a* fluorescence values on the O‒P phase from all field sites (**b**), as described in Methods.

In September, the plants from all elevations analyzed were characterized by clearly elevated fluorescence values (FL) at steps J and I, noticeable on the transient fluorescence curve of Chl *a*, compared to those in July (Figure 1a). In autumn, curves for shrub leaves growing at an elevation of 980 m a.s.l. and higher had a similar shape with a marked increase in FL intensity in the J‒I phase as compared to leaves from the lowest locality. The obtained JIP curves did not show the characteristic visible deflection for leaves at 568 m a.s.l. (Figure 1a—blue empty points). In the summer, differences in the so-called thermal phase (J‒P) were particularly visible between the highest and lowest localities and between 980 and 1320 m a.s.l. (Figure 1a).

The differential curves (ΔVt) of FL Chl *a* kinetics in the O‒P region for the localities (July and September measurements from all vegetation seasons) are shown in Figure 1b. The main differences between the summer–autumn period are visible in the J‒I phase. However, the biggest differences occurred in shrubs from the locality at 1215 m a.s.l., where the J‒band was higher and shifted in time (Figure 1b—green points).

**Figure 2:**
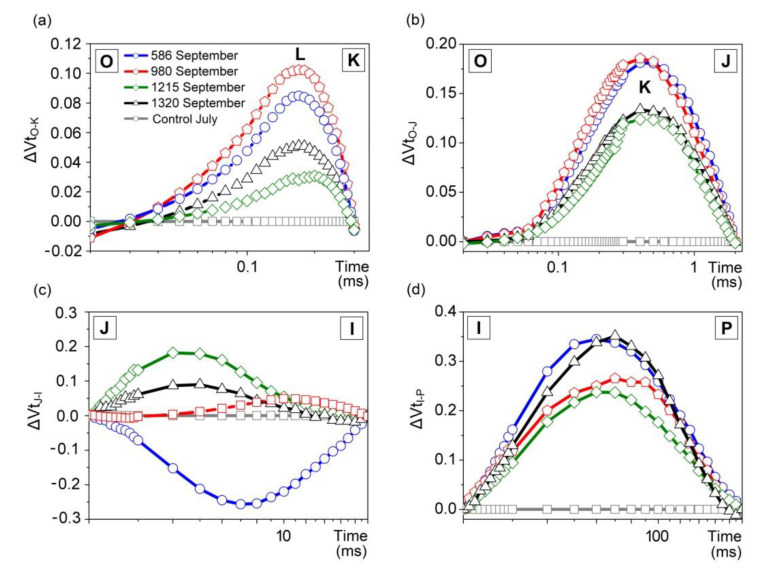
Differential fluorescence curves (ΔVt) of green alder (*Alnus viridis*) leaves for phases: (**a**) O‒K, (**b**) O‒J, (**c**) J‒I, and (**d**) I‒P, normalized to values corresponding to characteristic points in transitions of the chlorophyll fluorescence induction curve. The curves for individual sections were obtained by subtracting the Chl *a* fluorescence values recorded in July from the curves obtained in September. As a control for each field test stand, the curve obtained in July was adopted, as described in Methods.

The differential curves for the O‒K, O‒J, J‒I and I‒P phases were constructed analogously to ΔVt—as the difference between normalized FL values recorded in September and July, for each elevation (Figure 2). A detailed analysis of the O‒J phase revealed the presence of the K‒band (between 200 and 300 μs on Figure 2b), which was not visible on the O‒P integral differential curve (Figure 1b). In turn, the O‒K differential curve revealed the L‒band, illustrating the efficiency of photon absorption and the use of energy in the initial phase of the light reaction of photosynthesis. The L-band was present on differential curves in *A. viridis* leaves from all these localities (Figure 2a).

However, in plants growing at lower elevations, the L‒band reached values above 0.08 and was twice as high as in shrub leaves growing in the highest localities (1215 and 1320 m a.s.l.). A similar relationship was visible in the case of the K‒band (Figure 2b). In contrast to July, during September, some disturbances in the oxidative–reduction balance were visible in plants from field sites at 980, 1215, and 1320 m a.s.l. (positive values in the J‒I phase, Figure 2c). In the case of the lowest elevation, the reverse trend could be observed, as proven by the negative values ΔVt_J-I_. In turn, stress-typical negative bands did not appear in the I‒P region of the FL induction curve (Figure 2d).

**Figure 3:**
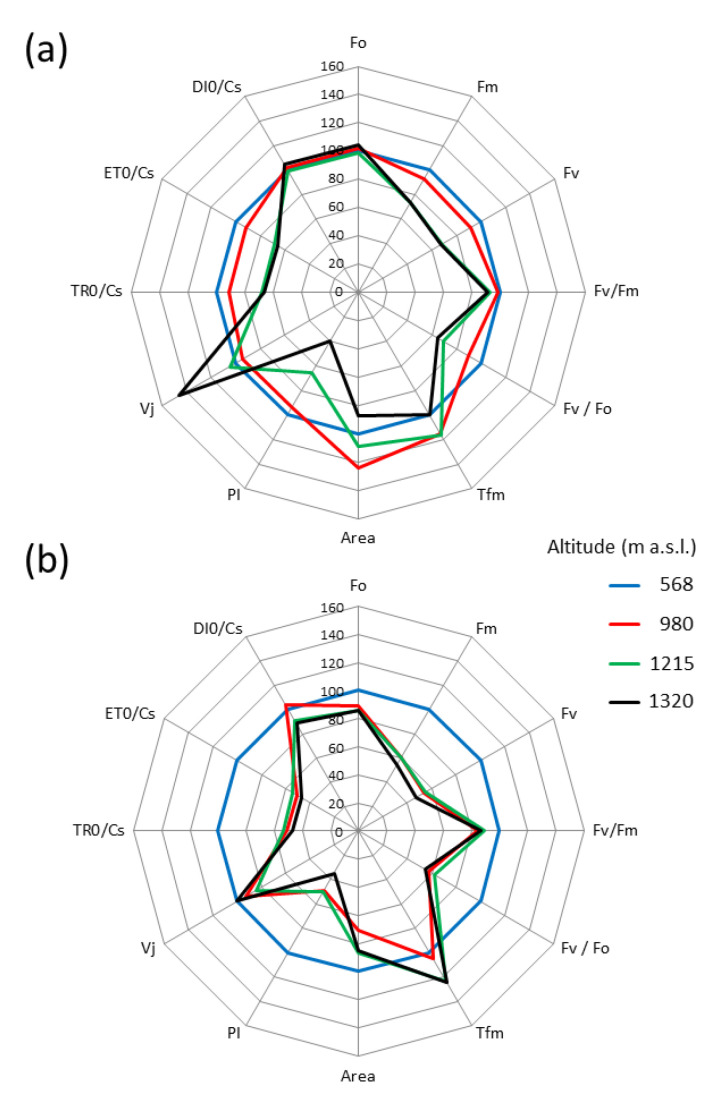
Values of selected Chl *a* fluorescence parameters of green alder (*Alnus viridis*) leaves (in % of control), recorded in (**a**) July and (**b**) September all field sites—in localities at elevations 980, 1215, and 1320 m a.s.l. The control was based on values from the lowest locality (586 m a.s.l.).

The chosen parameters of the fluorescence kinetics of Chl *a* are presented as a percentage deviation from the values obtained for *A. viridis* plants growing at the lowest locality (blue line—control) (Figure 3a,b). A comparison of the parameters of PSII photochemical efficiency in July in the control versus plants growing at 980 m a.s.l. did not show significant differences in the functioning of the photosynthetic apparatus (Figure 3a). However, leaves of *A. viridis* grown at 1215 m and 1320 m a.s.l. differed in term of the values of the Chl *a* fluorescence parameters when compared to the control. Among them, a significant decrease in the maximal fluorescence (F_m_), variable fluorescence (F_v_), efficiency of the water splitting complex on the PS II donor side (F_v_/F_0_), and the maximum quantum yield of PSII (F_v_/F_m_) was found (Figure 3a). With the increase in elevation, there was also a decrease in the rate of energy uptake and electron transport (TR0/Cs and ET0/Cs parameters, respectively), as well as decrease in the PSII performance index (PI) in relation to the control. A significant increase in the relative fluorescence value (Vj) in shrub leaves at an elevation of 1320 m a.s.l. was noticeable. The parameters values of plants growing at an elevation of 980 m a.s.l. were most similar to the control values (Figure 3a). Plants growing at positions 980, 1215, and 1320 m a.s.l. (in contrast to *A. viridis* growing at 586 m a.s.l.) show F_v_/F_m_ values lower than 0.83, which indicated a stress response of the photosynthetic apparatus. This differentiation was even greater for the PI parameter values, which better describes the physiological condition of the plant, than F_v_/F_m_. The PI value provides information on the number of active reaction centers per chlorophyll and initial reactions of the light phase with data on electron flux through RC [17] and is a good indicator of plant stress.

In September the Chl *a* fluorescence parameter values of plants growing at the highest localities (1215 and 1320 m a.s.l.) changed slightly (with the exception of Vj) compared to those from July (Figure 3b). The largest difference was only seen for *A. viridis* leaves at an elevation of 980 m a.s.l.

### 2.5. Reflectance of A. viridis Leaves

**Figure 4:**
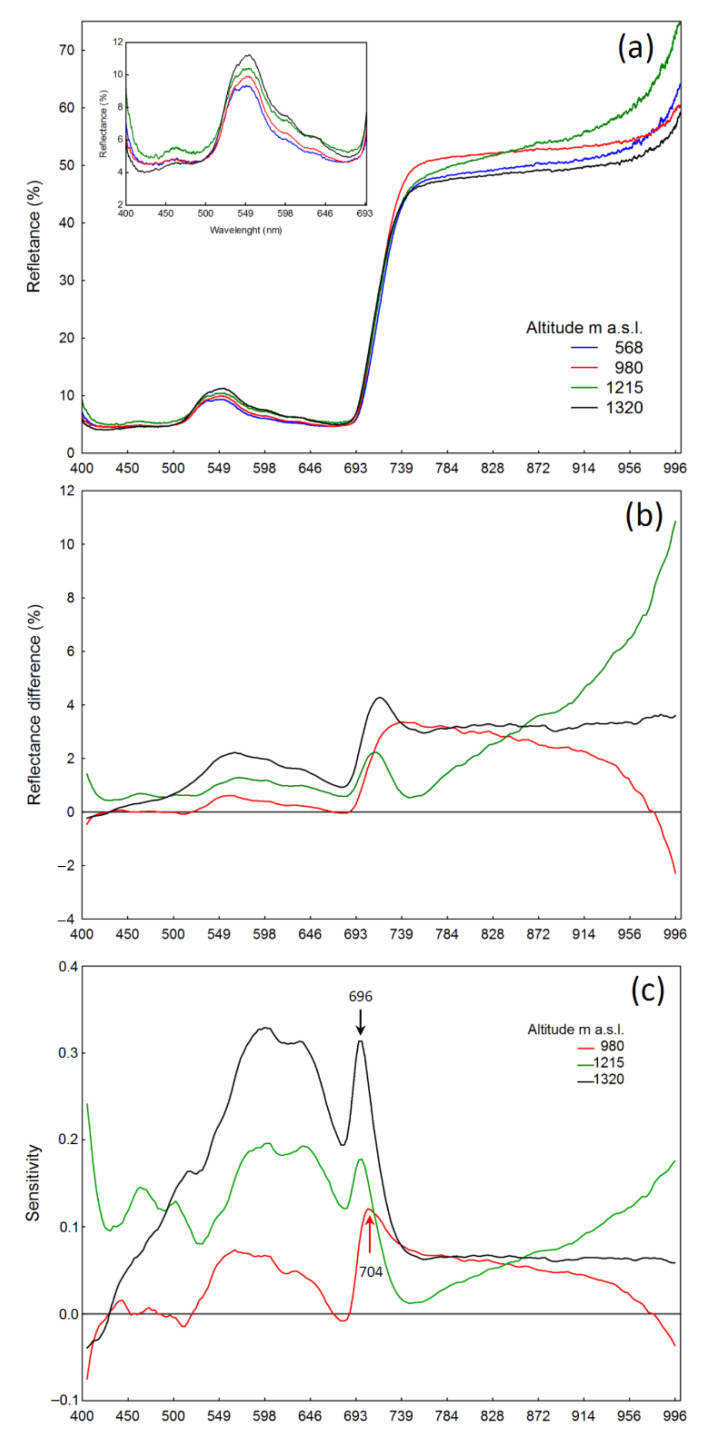
Reflectance curves for leaves of green alder (*A. viridis*) plants growing in the Bieszczady Mts. at different elevations: 568, 980, 1215, and 1320 m a.s.l. (**a**) reflectance—inset is an enlarged graph of PAR reflection; (**b**) reflectance difference—calculated by subtracting reflectance intensity values (%) for plants growing 568 m a.s.l. from reflectance intensity values for plants growing 980, 1215, or 1320 m a.s.l., respectively (see Material and methods); (**c**) sensitivity—calculated by dividing the RD values by mean reflectance values (%) for plants growing at 568 m a.s.l., *N* = 40.

The reflectance spectrum of *A. viridis* leaves in all localities analyzed was characterized by a similar curve shape (Figure 4). In terms of photosynthetically active radiation (PAR), the differences between the intensity of reflectance in plants at individual elevations were small. The reflectance intensity on the yellow–green spectrum, with its peak at 550 nm, increased with the locality elevation (Figure 4a—inside). The biggest difference was visible on the orange spectrum and at 696 nm for plants growing at 1215 and 1320 m a.s.l., when compared to the lowest locality (568 m a.s.l.). For a shrub at 980 m a.s.l. there was a shift in maximum sensitivity towards longer wavelengths (with a peak at 704 nm), with simultaneously lower sensitivity values on the yellow–orange spectrum. The minimum value was consistently around 670 nm for plants from all localities (Figure 4c).

An analysis of spectral reflectance indices showed that the lowest value of the anthocyanin reflectance index (ARI2) was found in plants at 568 m a.s.l. In the remaining localities, the ARI2 values were similar, although significantly higher than the values at 568 m a.s.l. (Table 4). The lowest carotenoid reflectance index (CRI1) was recorded in plants at 1215 m a.s.l. (Połonina Wetlińska). In the remaining localities (Łobozew, Przełęcz Wyżna, and Tarnica), the values of this parameter were more or less homogeneous, although significantly higher than those from Połonina Wetlińska. In the case of the flavonoid reflectance index (FRI), a significantly higher value, in comparison to remaining localities, was observed for shrubs growing at elevation of 1320 m a.s.l. (Tarnica). On the other hand, the value of the WBI coefficient for *A. viridis* leaves was lowest for shrubs growing at an elevation of 1215 m a.s.l. (Połonina Wetlińska) and deviated significantly from the values found at other localities. The highest photochemical reflectance index (PRI) value was recorded for plants growing at 568 m a.s.l., while the lowest PRI values were found in leaves of *A. viridis* at 1215 and 1320 m a.s.l. (Table 4).

## 3. Discussion

The significantly lower (compared to other localities) values for the leaf morphological parameters of *A. viridis* shrubs that grew on the highest, unprotected peak and sub-peak localities (1215 m and 1320 m a.s.l.) are most likely the result of the environmental stresses that these plants undergo (such as strong wind, large fluctuations of temperatures, and intensive solar irradiation) (Supplementary Figures S1 and S2). However, these values do not differ significantly from those given in the literature for 12 populations in the Polish Bieszczady Mountains and for four populations from Switzerland [18].

Low SLW values in the lower localities (568 m and 980 m a.s.l.) indicate that the large leaf area is accompanied by a low dry matter value (Table 1). To minimize the effects of stressors, plants usually develop leaves covered with a thick cuticle layer or trichomes. In the Bieszczady Mountains, lower localities are usually exposed to fewer strong wind and high temperature fluctuations, and are more likely to be shielded and shaded. Shading, for example, causes the plant to develop leaves with a large area, but thin leaf lamina [15,19]. The modifications in leaf morphology at the highest localities (at 1215 m and 1320 m a.s.l.) most likely result from various exposures to strong wind. The locality at 1215 m a.s.l. (Połonina Wetlińska) is positioned on a bare ridge, while the locality at 1320 m a.s.l. is found on a steep (about 45°) north-western slope below the peak of Tarnica. It seems, therefore, that exposure to stressful environmental factors related to a.s.l. locality influences the variability of the morphological parameters of *A. viridis* leaves.

The chlorophyll content in plant leaves and their respective proportions are controlled by many factors, both external and internal. The integrated activities of these factors determine the activity of the biosynthesis and degradation processes of Chl [20]. It was reported that in the leaves of *A. viridis* (the mountains of North Tyrol), the Chl content decreased with increasing elevation, with the highest content observed in July and the lowest in June and November. In the present study, no clear relationship was found between the height a.s.l. and the total Chl content. In terms of seasonal changes in the Chl content in *A. viridis* leaves, the Chl content in September was significantly lower compared to that in July only in the lower locations (568 m and 980 m a.s.l.). In this study, Chl content measurements in *A. viridis* leaves were conducted generally under lower elevations than in the Tyrolean mountains (650–1950 m a.s.l.). On the other hand, the differences in the seasonal Chl content might result from the fact that in lower positions, the radiation conditions vary more than in a peak locality. In lower localities, the leaves were thinner and had a larger surface area, which can result in a lower Chl content per unit area (Table 1 and Table 2).

Because the elevation of the localities did not significantly affect the Chl *a* content either in summer or autumn, the total content of Chl *a* + *b* reflected changes in the Chl *b* content at these localities (Table 2). In the early stages of plant growth, the leaf maximum Chl content was found to be controlled by phytochromes under optimum light conditions [21]. The red color of the light causes an increase in the total Chl content, and the quantitative composition of Chl *a* in relation to Chl *b* can change depending on the red to far-red ratio (R/FR). Red light usually favors the production of Chl *b*, which in turn leads to a decrease in the ratio of Chl *a*/*b*. In addition, the final value of this index under conditions of a high R/FR ratio (a lot of red) also depends on the age of the plant, and thus can vary within a large range, sometimes as much as from 1.0 to 2.2 in *Chenopodium rubrum* [21,22]. In fully exposed localities at an elevation of 1215 m and 1320 m a.s.l., the higher Chl *b* content in *A. viridis* leaves is probably due to the high R/FR ratio (Table 2). In contrast, the low Chl *b* content in *A. viridis* leaves observed in the locality 980 m a.s.l. in September is most likely the result of a fluctuation in the spectral composition of light at this study site during the growing season. In addition, at this locality, *A. viridis* grows in the vicinity of other plants.

It is assumed that δ^13^C values for C_3_ plants range from −20 to −35‰ (on average −27.5‰) [23,24]. In all localities, δ^13^C values oscillated near the average value, which indicates that these plants are able to bind CO_2_ directly, with the predominant involvement of the ribulose-1,5-bisphosphate carboxylase oxygenase (RuBisCO) enzyme (Table 3). However, the significantly less negative values of δ^13^C at an elevation of 1215 m a.s.l. indicate the least favorable vegetation conditions prevailing at this locality, which is most likely related to the presence of stress factors affecting the closing of stomata, especially drought. The significantly lower WBI values recorded in this locality confirm the water deficit conditions and appearance of leaf dehydration (Table 4).

Based on research conducted by [25], the relationship between carbon isotope discrimination and the intensity of water loss due to transpiration has been estimated. Therefore, it can be assumed that the δ^13^C values obtained may result from the fact that *A. viridis* shrubs growing in the studied localities differ in terms of their water use efficiency.

It is well known that discrimination of the ^15^N content in plants and soil occurs as a result of both biological and physicochemical processes [26,27]. Nevertheless, the processes conditioning the δ^15^N value in plants, and in the soil, are still poorly understood. In our study, high negative δ^15^N values correlated with high negative values δ^13^C (Table 3). The least negative values of δ^15^N (−1.13‰) were found in leaves in the locality 1215 m a.s.l., which at the same time were characterized by the lowest δ^13^C (−26.84‰). *A. viridis,* thanks to symbiosis with *F. alni* in root nodules, has the ability to bind N_2_ [5,6]. Initially, it was suggested that N isotope fractionation (^15^N discrimination) occurs during N_2_ binding in plant root nodules [28]. However, [29] demonstrated that during the N_2_ binding process, discrimination is rather minimal. Significant isotope fractionation may take place during subsequent transport and biochemical transformations that occur inside the host plant [29,30,31]. Unkovich et al. [29] also pointed out that root-nodule bacteria, which infect a symbiotic plant, have an effect on the fractionation of bound N in plant organs [32,33,34]. This was confirmed by studies on various *A. viridis* tissues in the Swiss Alps [9]. It cannot be ruled out that the difficult growth conditions resulting from the nature of the habitat reduce the internal discrimination of ^15^N.

The increase in FL at steps J and I on the Chl *a* fluorescence curve, as observed for all measurements in September, suggests a lower number of electron transporters on the PSII acceptor side than in July. The shape of the induction curve and the relatively high FL intensity in the J‒I phase in plants at 980, 1215, and 1320 m a.s.l. indicates deviations from the oxidation–reduction balance of the Q_A_ pool (Figure 1a), as reported before by [35]. The I–P phase illustrates the photosystem I (PSI) acceptor side reduction process [36]. The slightly lower FL intensity in the I‒P phase in the localities at 980 and 1215 m, specifically visible in autumn, suggests a lower efficiency in the reduction of electron transporters by terminal electron acceptors such as ferredoxin, NADP^+^, and RuBP as indicated also by [11]. An analysis of the J‒P phase shows that in July, the highest efficiency of electron transport from Q_A_ to PSI was demonstrated by plants in the locality at 980 m a.s.l., which may indicate that the photosynthetic apparatus is more efficient and better acclimated to environmental stress (Figure 1a—red filled points) [37,38]. High FL values in the thermal phase J-P part of the JIP curve are related to the reduction of the final steps of the electron transport chain [39]. In both July and September, the shape of the curves for shrub leaves in the locality at 568 m a.s.l. suggests that of the localities analyzed, this is where the photosynthetic apparatus of *A. viridis* plants is in the best condition. Differences between individual habitats in the summer–autumn period (ΔVt) correspond to the degree of reduction in the secondary electron acceptor (Q_B_), plastochinone (PQ), and cytochrome b_6_f [14] and are visible in the J‒I phase (Figure 1b). The highest value of ΔVt was observed in plants growing at an elevation of 1215 m a.s.l., which points to a strong reaction of *A. viridis* photosynthetic apparatus to the unstable ambient conditions prevailing at this locality.

The disclosure of the K and L–bands on the differential FL curves indicates disturbances during the course of the photosynthetic reaction in *A. viridis* leaves at the end of the growing season. In the autumn, the plants in all localities decreased the efficiency of energy transfer between the antenna complexes and the PSII reaction center, as evidenced by the appearance of the L‒band on the O‒K differential curve (Figure 2a). *A. viridis* shrubs in localities situated at 980 m a.s.l. (and below) showed greater disorder (higher L‒band and K‒band values) in the initial reactions of the light phase than those growing at higher altitudes. This is most likely related to the impairment of the PSII donor side, in particular, the partial oxygen-evolving-complex deactivation [40]. The lower ΔVt value for the L and K bands in shrub leaves at higher localities is possibly the result of photosynthetic apparatus acclimation to the more difficult environmental conditions prevailing there throughout the year. Presumably, the decrease in the intensity of Chl *a* FL in the L-band is associated with an increase in energy transport efficiency between the light harvesting complex of PSII (LHCII) and the PSII reaction center [36]. The positive values for bands visible on the J‒I differential curve in the higher localities are evidence of a balanced disorder between Q_A_ reduction and oxidation (Figure 2c). The response of plants to unfavorable environmental conditions often results in negative values in the I‒P region of the FL induction curve [41]. However, in *A. viridis* leaves, there were no bands characteristic of stress symptoms in spite of the slight decrease in the number of available NADP molecules in the PSI reaction center (Figure 2d). This observation indicates that the long-term effect of possible multi-stress is not reflected in the I‒P phase. The close correlation between PSII function and Chl *a* fluorescence parameters allows a quick and precise evaluation of the photosynthesis response to environmental changes [39,42]. In September, in *A. viridis* shrubs growing at an elevation of 980 m a.s.l., the values of majority of the PS II photochemical efficiency coefficients (except for the time necessary to obtain maximum fluorescence (T_fm_)) approached those observed at sub-peak and peak localities (1215 and 1320 m a.s.l.). For healthy plants under stress-free conditions, the maximum value of the F_v_/F_m_ coefficient is about 0.83 [43]. An F_v_/F_m_ value less than 0.83 indicates that the plant has been exposed to stress factors affecting the functioning of PSII and is considered to be a sensitive indicator of PSII photochemical efficiency reduction [44]. Miszalski et al. [45] demonstrated that in *Picea abies* in the Tatra Mountains, increasing elevation decreased the F_v_/F_m_ and F_m_ values. However, in our research, the leaves of *A. viridis* shrubs were less differentiated in terms of the F_v_/F_m_ parameter than PI. Consequently, in *A. viridis* leaves, PI values better reflect changes in the photochemical activity of PSII in response to changing environmental conditions (Figure 3a,b). The Chl *a* fluorescence parameter changes and differences in FL intensity in the J‒I phase were similar to previous reports, which indicates that as the elevation increases, the level of environmental stress usually increases. This additional stress is probably associated with a drop in the average annual temperature and air humidity, higher solar radiation intensity, and a shortage of nutrients in the soil [14,38,41]. As the elevation increases, the daily amplitudes of environmental parameters increase, as does the role of wind [10].

An analysis of leaf reflection allows for a non-destructive and quick assessment of plant sensitivity to the effects of stress. An increase in reflection values in the photosynthetically active radiation (PAR) range, with its peak in the green spectrum, usually occurs under stressful conditions [46]. In the present study, we observed changes in the reflection intensity of *A. viridis* leaves as a function of increasing elevation a.s.l.—it was clearly greater at the highest localities. This was reflected in the peak heights found on the sensitivity plots. Despite the slight differences in the reflection intensity (Figure 4a), the sensitivity values had clear visible peaks, characteristic of a stress response (Figure 4c). The maximum values of the sensitivity were observed at the highest localities (1320 and 12,315 m a.s.l.). Bands at the same wavelengths were also observed on the sensitivity curves for other plant species subjected to drought stress, fungal infection, strong dehydration, or high-light stress [47,48,49]. For leaves of *A. viridis* that grow on Połonina Wetlińska (1215 m a.s.l.), an intense reflection was additionally observed in the far-red and the near infra-red (NIR) range (starting at 830 nm) compared to the other localities studied. This is probably the result of the lower hydration of leaves in this locality; this assumption was confirmed by the low WBI (Table 4).

Of the substances responsible for absorbing harmful UV radiation, flavonoids, anthocyanins, and carotenoids play the most important role [10]. Analyzing reflectance indices of light radiation from leaves in relation to the shrub study site is a valuable source of information about the changes in the content of plant pigments. The lowest amount of anthocyanins in *A. viridis* leaves (ARI2 index) was observed in the lowest growth locality (Table 4). In other populations analyzed, the content of anthocyanins was comparable. The carotenoid content, estimated on the basis of the CRI1 index, was lowest in *A. viridis* leaves on Połonina Wetlińska (1215 m a.s.l.). The highest flavonoid content was demonstrated for shrubs growing on Tarnica, at 1320 m a.s.l. The small number of flavonoids and carotenoids in the *A. viridis* leaves on Połonina Wetlińska (low values of the FRI and CRI1 coefficient, Table 4) is somewhat surprising, taking into account that these pigments are protective against UV radiation. An explanation for this fact may be the high anthocyanin content of these leaves, which may provide them sufficient protection against the harmful effects of radiation. The WBI values for different plant species usually range from 0.8 to 1.2 [50]. The WBI values obtained for *A. viridis* (about 0.9), therefore indicate relatively good hydration of plant tissues in the localities studied (Table 4). Even the lowest hydration level, which occurred in the leaves of plants growing at an elevation of 1215 a.s.l., can be considered to be within the norm. In turn, the PRI coefficient is correlated with zeaxanthin (de-epoxidation in the xanthophyll cycle) and the effectiveness of PAR utilization by plants [51]. Higher PRI values indicate better PAR utilization efficiency. Gamon et al. [52] showed that, using the PRI, it is possible to track changes in the effectiveness of light radiation use in the photosynthetic process of plants affected by various environmental factors (e.g., the availability of mineral substances). The best use of PAR by *A. viridis* was recorded in the lowest field position, at 568 m a.s.l., which may be the result of growth under near-optimal conditions.

## 4. Material and Methods

### 4.1. Study Sites and Plant Material

The studies were carried out on green alder, *Alnus viridis* (Chaix) DC., leaves in the years 2014–2016. Research was conducted at the beginning and at the end of their growing season, i.e., in July and September, respectively. Four localities were chosen at different elevations: three in the Bieszczady National Park (BNP, south-eastern part of Poland) and one located outside its borders (Supplementary Table 1, Figures S1 and S2). Of the localities in the area of BNP, the highest is located on a steep slope (with an angle of inclination of about 45°) below the peak of Tarnica (1320 m a.s.l.). It is considered the most natural habitat and it is the highest habitat of *A. viridis* in Poland. A dense and quite extensive community of alder bushes is found there. The next locality is situated on the summit of Połonina Wetlińska (grassland—1215 m a.s.l.), above the upper forest border in the BNP meadows. *A. viridis* grows there as a “single—crumbled” shrub in open space on the ridge or in other lower pasture vegetation. The last locality within BNP is located on a mountain pass called Przełęcz Wyżna (980 m a.s.l.) (Supplementary Table S1). *A. viridis* is found there on the edge of the forest, in close proximity to other bushes and tall trees, creating compact clusters. The locality outside the BNP is also the lowest, in post-agricultural areas, near the village Łobozew (568 m a.s.l.). The shrubs are larger there, in terms of their morphology, than those growing on the Przełęcz Wyżna and on Połonina Wetlińska (Supplementary Figures S1 and S2).

Analyses were performed on intact leaves (directly on a shrub) as well as leaves detached and collected from *A. viridis* shrubs in the July and September months. Measurements and leaf collection were performed only on sunny days with a clear sky and 4–6 h after the beginning of the light period. Leaves were removed from various parts of the bush under comparable light conditions. For further study, usually 20 similar leaves per plant for one measurement were used/collected—in all localities over the course of two consecutive days. Plant material was collected and kept in a liquid nitrogen container for transport and analyses. The morphological variability of the leaves and the reflection of light radiation was analyzed in September. Leaves for ^13^C and ^15^N discrimination analyses were collected only in September. The September measurements made it possible to assess the course of physiological processes taking place at the height of the growing season and reflected the “physiological history” of the *A. viridis* leaves.

### 4.2. Leaf Morphology Parameters

Leaf shape was analyzed using the computer image analysis device WinDIAS_3, (GEOMOR—TECHNIK, Szczecin, Poland). Leaf parameters such as length, width, perimeter, and surface area were determined. Specific leaf weight (SLW) was calculated by dividing the dry weight of the leaf (mg) by its surface area (cm^2^). The results presented in the paper are mean values from measurements made on 60 selected leaves from each locality (described above).

### 4.3. Chlorophyll Content in Leaves

The Chl content of *A. viridis* leaves was measured according to the method described by [53]. At each locality, two discs with an area of 1.76 cm^2^ were cut from 10 selected similar leaves, by corkscrew, symmetrically on both sides of the primary nerve (midrib); these samples were placed immediately in test tubes containing 5 mL dimethyl sulfoxide (DMSO). After transfer to the laboratory, the tubes were heated for 3 h at 65 °C to extract Chl. The extract was then poured into spectrophotometric cuvettes, and the absorbance was measured using a CE2501 spectrophotometer, 2000 series (Cecil Instruments Ltd., Cambridge, UK) at λ = 665 and 648 nm.

The contents of Chl *a* and *b* were calculated according to the following formulas:

$$\mathrm{Chl}\;a\;\mathrm{content}=\frac{\left(14.85\;\times A_{665}-5.14\;\times A_{648}\right)\times V}{1000\times W}\;\mathrm{Chl}\;b\;\mathrm{content}=\frac{\left(25.48\;\times A_{648}-7.36\;\times A_{665}\right)\times V}{1000\times W}\;\mathrm{Chl}\;a+b\;\mathrm{content}=\frac{\left(7.49\;\times A_{665}-20.34\;\times A_{648}\right)\times V}{1000\times W}$$

where A—absorbance at λ = 665 or 648 nm; V—volume of extract (cm^3^); W—sample surface area (cm^2^).

### 4.4. The Content of Carbon Isotope ^13^C and Nitrogen ^15^N

The lyophilized leaves of *A. viridis*, harvested at the end of the growing seasons (20 leaves from each locality), were ground to powder in an agate mortar. The stable carbon and nitrogen isotopes were analyzed on a Thermo Flash EA 1112HT elemental analyzer coupled to a Thermo Delta V Advantage mass spectrometer (Thermo Fisher Scientific, Bremen, Germany) in the continuous flow system. The samples wrapped in tin foil were burnt in an oxygen atmosphere at 1020 °C. The CO_2_ and N_2_ obtained from the combustion were separated on a chromatographic column and directly inserted into the spectrometer with a capillary tube. Measurements were calibrated via international standards USGS 40, USGS 41, and IAEA 600 [54]. The ^13^C results are shown in δ form relative to the VPDB standard. The ^15^N results were given in δ form relative to the N_2_ standard.

### 4.5. Chl a Fluorescence Measurements

Chl *a* fluorescence kinetic parameters were analyzed using the Handy-PEA portable fluorimeter (Hansatech Instruments, Norfolk, UK), allowing for non-invasive measurements, according to [55]. Each time, measurements were made on the upper surface of 20 selected leaves. Before Chl *a* fluorescence measurements (actinic light 3 mmol (quantum) m^−2^ s^−1^), leaves were acclimated to darkness for 20 min using the measuring clips. Results were read and analyzed using the PEA Plus (Hansatech Instruments, Norfolk, UK) and Microsoft Excel 2010 software. The following parameters were analyzed: minimum fluorescence (F_0_), F_m_, F_v_, F_v_/F_m_, F_v_/F_0_, T_fm_, surface area above the chlorophyll fluorescence induction curve (Area), PI, V_J_, TR_0_/Cs, ET_0_/Cs, and DI_0_/Cs. These data were used to perform the JIP test, taking the following fluorescence intensity measurement points: O—20 μs, J—200 μs, I—30 ms, and P—300 ms. The curves of fluorescence kinetics (ΔVt) were obtained by subtracting the normalized fluorescence values between the points O‒P, measured in each field site in September from those recorded in July (accepted as a control), according to the formula:

ΔVt = Vt_IX_ − Vt_VII_

where Vt_IX_—relative variable fluorescence in September; Vt_VII_—relative variable fluorescence in July

### 4.6. Reflection of Radiation from the Leaves

The reflectance was measured each time on the upper side of the leaf blade of selected leaves using a miniature leaf spectrometer CI-710 (CID Bio—Science, Camas, WA, USA). The presented results are mean values from measurements taken on fully developed leaves from two growing seasons. The signal integration time was 350 ms; the signal smoothing factor (boxcar) was set to 10. Each measurement is the average of 10 scans. Based on the results, the reflectance intensity curves as a function of the wavelength of light were plotted. Reflectance coefficients were calculated automatically by the instrument on the basis of the following formulas: ARI2 = (R^−1^_550_ − R^−1^_700_) ∙ R_800_ [56]; CRI1 = (R^−1^_520_ − R^−1^_700_) ∙ R_800_ [56]; FRI = (R^−1^_410_ − R^−1^_460_) ∙ R_800_ [56]; WBI = R_970_ ∙ R_900_^−1^ [57]; PRI = (R_531_ − R_570_) ∙ (R_531_ + R_570_)^−1^ [52].

R is the intensity of reflectance at the wavelength (in nm) given in the index.

The reflectance difference and sensitivity allow for the identification of wavelength, at which the reflectance value changes most, under the influence of environmental stress [58]. The reflectance difference (RD) was calculated according to the formula given by Carter et al. [47]:

RD = R_X_ − R_568_

where R_X_—reflectance intensity (%) between 400 and 1000 nm in plants growing at 980, 1215, or 1320 m a.s.l., strongly exposed to the effect of multistress; R_568_—reflectance intensity (%) between 400 and 1000 nm in plants growing at 568 m a.s.l.; this was used as the reference group.

The sensitivity was calculated by dividing the RD values by the mean reflectance values (%) for plants growing at 568 m a.s.l., according to the formula: sensitivity = RD (R_568_)^−1^.

### 4.7. Statistical Analysis

All data are mean values from measurements made on leaves in individual localities during the growing seasons (2014–2016). The significance of the differences between the averages was examined using one-way ANOVA. The significance of differences between the means was compared using the Tukey test for different N at a level of *p* ≤ 0.05. The results were statistically analyzed and the Chl fluorescence curves were normalized using the StatSoft, Inc. computer program (2014) STATISTICA (data analysis software system, version 12, www.statsoft.com).

## 5. Conclusions

Green alders growing in positions below the upper forest line generally show better physiological parameters than plants growing in higher localities which is associated with less environmental stress. Thanks to its multiple physiological attributes, such as efficient vegetative reproduction and the ability to perform symbiotic nitrogen fixation, *A. viridis* can rapidly take over new habitats, displacing indigenous plant species from lowland ecosystems. The natural occurrence of this taxon in BNP is above the upper limit of the forest. However, recently it has occupied lower positions, mostly in secondary post-farming areas. The growing range of *A. viridis* to the north and west indicates that this species may become extensive in Poland.

## Figures and Tables

**Table 1:** Morphological parameters of the leaves of green alder (*Alnus viridis*) growing in the Bieszczady Mts. at various elevations a.s.l.

Leaf Morphological Parameters	Elevation (m a.s.l.)			
	568	980	1215	1320
Surface area (cm^2^)	11.17 ± 0.69 a	9.46 ± 0.42 b	6.63 ± 0.66 c	6.31 ± 0.26 c
Length (cm)	4.65 ± 0.11 a	4.24 ± 0.08 b	3.50 ± 0.12 c	3.55 ± 0.07 c
Width (cm)	3.45 ± 0.10 a	3.12 ± 0.08 b	2.49 ± 0.09 c	2.52 ± 0.06 c
Perimeter (cm)	14.21 ± 0.44 a	13.04 ± 0.34 b	11.16 ± 0.50 c	11.44 ± 0.27 c
SLW coefficient (mg_DW_ cm^−2^)	7.94 ± 1.17 d	9.43 ± 0.31 c	14.11 ± 0.34 a	13.24 ± 1.28 b

Values marked with different letters differ significantly at *p* ≤ 0.05 according to Tukey’s test, *N* = 40. Mean values ± SD.

**Table 2:** Chlorophyll content in the leaves of green alder *(Alnus viridis*) collected in July and September from plants growing in the Bieszczady Mts. at various elevations a.s.l.

Chlorophyll Content	Elevation (m a.s.l.)							
	568		980		1215		1320	
	July	September	July	September	July	September	July	September
Chl *a* (mg cm^−2^)	0.062 ± 0.0006 a	0.040 ± 0.001 b	0.062 ± 0.0002 a	0.041 ± 0.006 b	0.063 ± 0.0002 a	0.041 ± 0.0002 b	0.062 ± 0.0006 a	0.041 ± 0.0004 b
Chl *b* (mg cm^−2^)	0.027 ± 0.0013 d	0.039 ± 0.002 c	0.054 ± 0.0016 ab	0.036 ± 0.002 cd	0.047 ± 0.002 bc	0.065 ± 0.002 a	0.040 ± 0.0025 c	0.057 ± 0.003 a
Chl *a* + *b* (mg cm^−2^)	0.090 ± 0.002 d	0.079 ± 0.002 e	0.116 ± 0.001 a	0.076 ± 0.007 e	0.109 ± 0.002 ab	0.107 ± 0.002 abc	0.103 ± 0.003 bc	0.098 ± 0.003 cd
Chl a/*b*	2.281 ± 0.093 a	1.040 ± 0.041 d	1.151 ± 0.036 cd	1.178 ± 0.118 cd	1.368 ± 0.054 bc	0.641 ± 0.025 e	1.601 ± 0.094 b	0.749 ± 0.048 e

Values marked with different letters differ significantly at *p* ≤ 0.05 according to Tukey’s test, *N* = 40. Mean values ± SD.

**Table 3:** Isotopic composition of carbon (^13^C) and nitrogen (^15^N) in the leaves of green alder *(Alnus viridis*) collected in September from plants growing in the Bieszczady Mts. at various elevations a.s.l.

Elevation (m a.s.l.)	δ^13^C (‰)	δ^15^N(‰)
568	−30.78 ± 0.03 d	−2.00 ± 0.006 d
980	−28.12 ± 0.02 c	−1.88 ± 0.006 c
1215	−26.84 ± 0.02 a	−1.13 ± 0.009 a
1320	−27.97 ± 0.02 b	−1.42 ± 0.005 b

Values marked with different letters differ significantly at *p* ≤ 0.05 according to Tukey’s test, *N* = 40. Mean values ± SD.

**Table 4:** Spectral reflectance indices for green alder *(Alnus viridis*) leaves growing in the Bieszczady Mts. at various elevations a.s.l.

Elevation (m a.s.l.)	Reflectance Indices				
	ARI2	CRI1	FRI	WBI	sPRI
568	0.239 ± 0.030 b	0.076 ± 0.002 a	−0.659 ± 0.090 b	0.919 ± 0.009 b	0.058 ± 0.002 a
980	0.540 ± 0.063 a	0.089 ± 0.004 a	−0.235± 0.065 b	0.958 ± 0.005 a	0.038 ± 0.006 b
1215	0.455 ± 0.057 a	0.065 ± 0.003 b	−0.701 ± 0.096 b	0.872 ± 0.006 c	0.020 ± 0.004 c
1320	0.460± 0.041 a	0.088 ± 0.002 a	0.376 ± 0.182 a	0.937 ± 0.005 ab	0.011± 0.005 c

Values in individual columns, marked with different letters, differ significantly at *p* ≤ 0.05 according to Tukey’s test, *N* = 40. Mean values ± SD.

## Data Availability

All data are contained within the article.

## Supplementary Materials

The following are available online at https://www.mdpi.com/2223-7747/10/1/96/s1 Table S1: Summary of test stands with geographical coordinates and altitude above sea level, Figure S1: Study sites of the *Alnus viridis* (Chaix) DC. on the map. Green colour—the border of the Bieszczady National Park (BNP); blue colour—the border of buffer zone of the BNP; white colour—state borders, Figure S2: Study sites of the *Alnus viridis* (Chaix) DC. in the Bieszczady National Park (BNP) and buffer zone.

## Abbreviations

Area, surface area above the chlorophyll fluorescence induction curve; ARI2, Anthocyanin Reflectance Index; a.s.l., above sea level; BNP, Bieszczady National Park; CRI1, Carotenoid Reflectance Index; Cs, illuminated cross-section of a leaf sample; DI_0_/Cs, dissipated energy flux per cross-section of a sample; ET_0_/Cs, electron transport flux per cross-section; F_0_, minimum fluorescence; F_m_, maximum fluorescence; FL, fluorescence; FRI, Flavonol Reflectance Index; F_v_, variable fluorescence (F_m_–F_0_); F_v_/F_0_, maximum efficiency of the water-splitting reaction of the donor side of PSII; F_v_/F_m_, maximum quantum yield of PSII of a dark-adapted sample; LHCII, Light-Harvesting Complex of PSII; NIR, Near Infra-Red; JIP, chlorophyll *a* fluorescence JIP transient; PAR, Photosynthetically Active Radiation; PI_abs_, PSII performance index; RD, Reflectance difference; sPRI, scaled Photochemical Reflectance Index; PSI, Photosystem I; PSII, Photosystem II; Q_A_, plastoquinone Q_A_; R/FR, Red/Far red; RuBisCO, ribulose-1,5-bisphosphate carboxylase oxygenase; SLW, Specific Leaf Weight; T_fm_, time necessary to obtain maximum fluorescence; TR_0_/Cs, Trapping flux leading to Q_A_ reduction per cross-section (CS); WBI, Water Band Index; Vj, relative variable fluorescence at J–step; Vt, relative variable fluorescence.
