# Supplementary material for: Ecophysiological Variability of Alnus viridis (Chaix) DC. Green Alder Leaves in the Bieszczady Mountains (Poland)
Source: Plants (Basel). 2021 Jan 6;10(1):96. doi: 10.3390/plants10010096 (PMC7825132; doi:10.3390/plants10010096)
Supplement: Supplementary file 1 [file plants-10-00096-s001.pdf]

Supplementary material

Table S1. Summary of test stands with geographical coordinates and altitude above sea level.

Position/Study site (geographical name)	Geographical location	Elevation (m a.s.l.)
Łobozew	N 49° 23’ 31.24” E 22° 31’ 32.24”	568
Przełęcz Wyżna	N 49° 08’ 56.80” E 22° 33’ 04.47”	980
Połonina Wetlińska	N 49° 09’ 36.68” E 22° 32’ 43.38”	1215
Tarnica	N 49° 04’ 35.38” E 22° 43’ 34.50”	1320

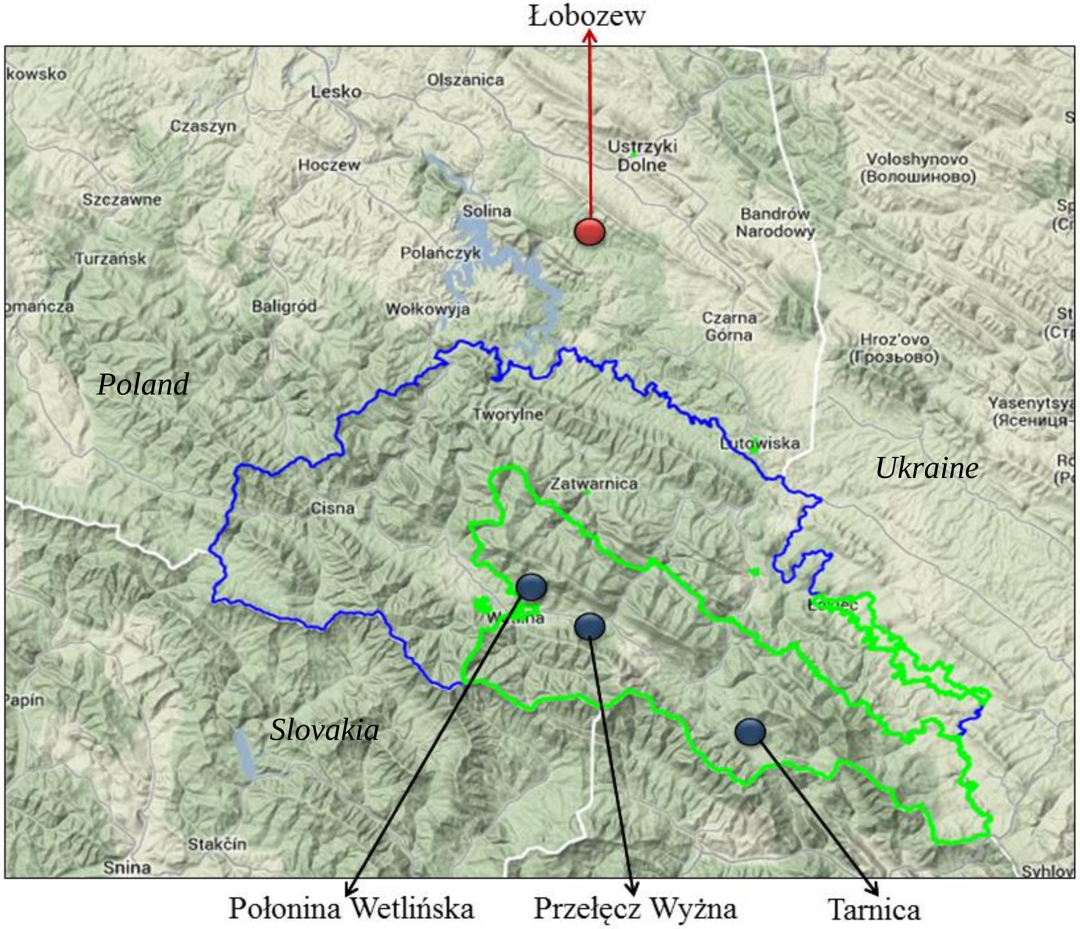

Figure S1. Study sites of the *Alnus viridis* (Chaix) DC. on the map. Green colour – the border of the Bieszczady National Park (BNP); blue colour – the border of buffer zone of the BNP; white colour – state borders.

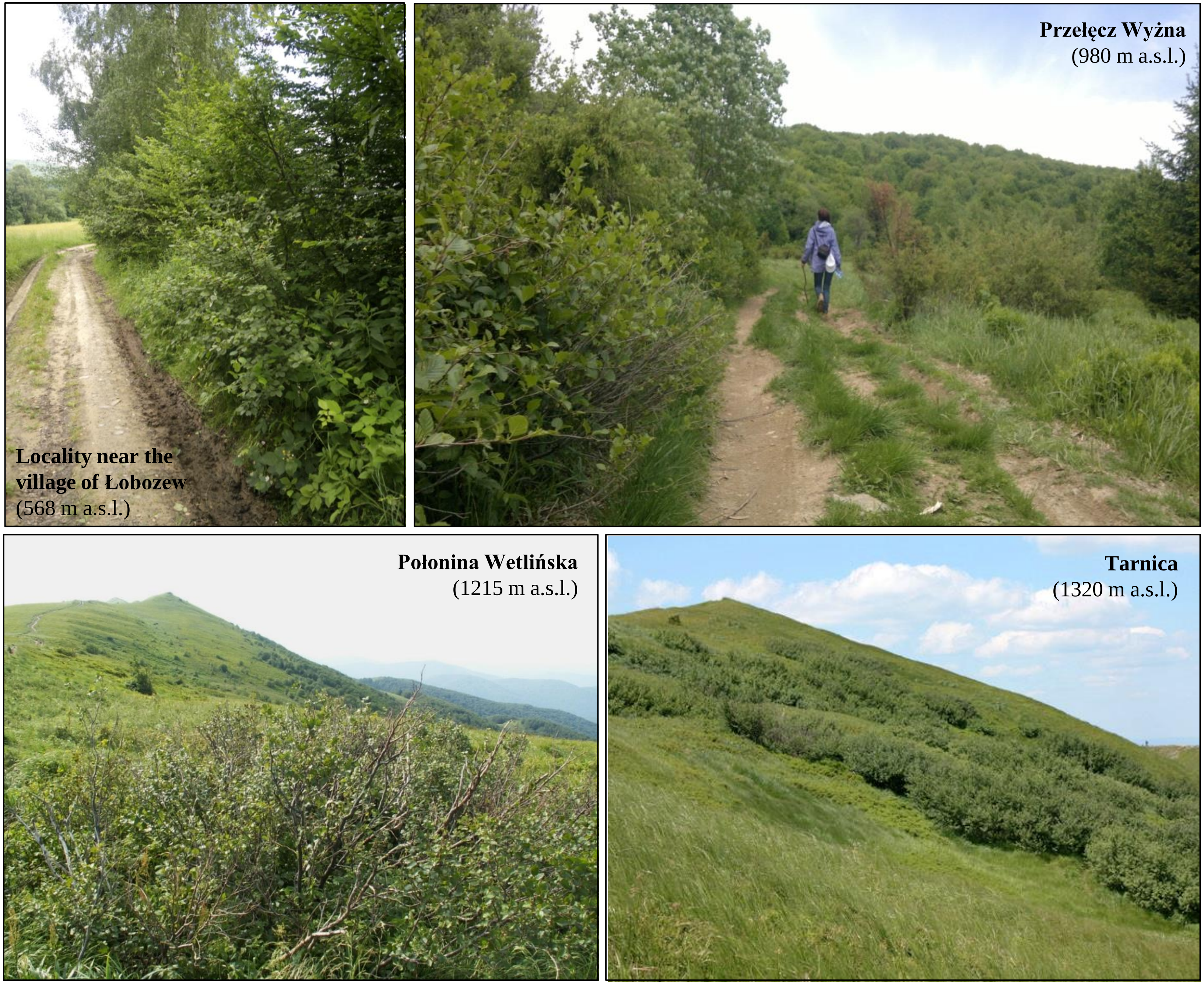

Figure S2. Study sites of the *Alnus viridis* (Chaix) DC. in the Bieszczady National Park (BNP) and buffer zone.
